# Antimicrobial resistance in urinary pathogens and culture-independent detection of trimethoprim resistance in urine from patients with urinary tract infection

**DOI:** 10.1186/s12866-022-02551-9

**Published:** 2022-05-24

**Authors:** Yinka M. Somorin, Nichola-Jane M. Weir, Sally H. Pattison, Martin A. Crockard, Carmel M. Hughes, Michael M. Tunney, Deirdre F. Gilpin

**Affiliations:** 1 School of Pharmacy, Queen’s University Belfast, 97 Lisburn Road, Belfast, Northern Ireland; 2 Wellcome-Wolfson Institute for Experimental Medicine, Queen’s University Belfast, 97 Lisburn Road,, Belfast, Northern Ireland; 3 Randox Laboratories Ltd, 55 The Diamond Road, Crumlin, Northern Ireland

**Keywords:** Trimethoprim resistance, Urinary tract infection, *dfrA*, Rapid detection, Antimicrobial prescription

## Abstract

**Background:**

Although urinary tract infections (UTIs) are extremely common, isolation of causative uropathogens is not always routinely performed, with antibiotics frequently prescribed empirically. This study determined the susceptibility of urinary isolates from two Health and Social Care Trusts (HSCTs) in Northern Ireland to a range of antibiotics commonly used in the treatment of UTIs. Furthermore, we determined if detection of trimethoprim resistance genes (*dfrA*) could be used as a potential biomarker for rapid detection of phenotypic trimethoprim resistance in urinary pathogens and from urine without culture.

**Methods:**

Susceptibility of *E. coli* and *Klebsiella* spp. isolates (*n* = 124) to trimethoprim, amoxicillin, ceftazidime, ciprofloxacin, co-amoxiclav and nitrofurantoin in addition to susceptibility of *Proteus mirabilis* (*n* = 61) and *Staphylococcus saprophyticus* (*n* = 17) to trimethoprim was determined by ETEST® and interpreted according to EUCAST breakpoints. PCR was used to detect *dfrA* genes in bacterial isolates (*n* = 202) and urine samples(*n* = 94).

**Results:**

Resistance to trimethoprim was observed in 37/124 (29.8%) *E. coli and Klebsiella* spp. isolates with an MIC_90_ > 32 mg/L. *DfrA* genes were detected in 29/37 (78.4%) trimethoprim-resistant isolates. Detection of *dfrA* was highly sensitive (93.6%) and specific (91.4%) in predicting phenotypic trimethoprim resistance among *E. coli and Klebsiella* spp. isolates. The *dfrA* genes analysed were detected using a culture-independent PCR method in 16/94 (17%) urine samples. Phenotypic trimethoprim resistance was apparent in isolates cultured from 15/16 (94%) *dfrA*-positive urine samples. There was a significant association (*P* < 0.0001) between the presence of *dfrA* and trimethoprim resistance in urine samples containing Gram-negative bacteria (Sensitivity = 75%; Specificity = 96.9%; PPV = 93.8%; NPV = 86.1%).

**Conclusions:**

This study demonstrates that molecular detection of *dfrA* genes is a good indicator of trimethoprim resistance without the need for culture and susceptibility testing.

**Supplementary Information:**

The online version contains supplementary material available at 10.1186/s12866-022-02551-9.

## Introduction

Urinary Tract Infections (UTIs) are among the most common bacterial infections that occur in primary care [1] and the second most common reason for prescription of antibiotics in England [2]. Treatment of UTI is most commonly empirical, based on clinical suspicion and/or a positive urine dipstick test. Although urine dipstick tests are rapid and can be used at the point of care, their value is primarily in their ability to rule out rather than confirm infection [3, 4]. Where further analysis of a urine sample is required, the presence of a uropathogen is established by microscopy, culture and subsequent antimicrobial susceptibility testing (AST). However, as conventional culture and AST may take up to 72 h, commencement of appropriate treatment may be delayed. This could lead to clinical complications as well as longer and more frequent hospitalization of patients such as the elderly and those who are immunocompromised [5].

Trimethoprim is currently only recommended as first-line treatment for UTIs if there is a low risk of resistance with nitrofurantoin, the antibiotic of choice [6]. However, patients prescribed nitrofurantoin frequently present with gastrointestinal side effects. Moreover, nitrofurantoin is not recommended for use in patients with poor renal function, defined as an estimated glomerular filtration rate < 45 ml/minute, which is common amongst the elderly. Therefore, trimethoprim remains the preferred choice for treatment of many UTIs. Gram-negative bacteria, particularly *E. coli* and *Klebsiella* spp., are the most commonly isolated uropathogens with high levels of trimethoprim resistance observed [7]. Resistance to trimethoprim was 39% in *E. coli*, 26.7% in *Klebsiella* spp. and 41.9% in *Proteus mirabilis* whereas resistance to nitrofurantoin was 4% in *E. coli,* 34.8% in *Klebsiella* spp. and 0% in *Proteus mirabilis* [8, 9]. In contrast, resistance is rare in *Staphylococcus saprophyticus* [10]. This study determined the susceptibility of urinary *E. coli, Klebsiella* spp., *Proteus mirabilis* and *Staphylococcus saprophyticus* isolates to a range of antibiotics commonly used in the treatment of UTIs. We also determined if there was an association between the prevalence of *dfrA* genes, conferring resistance to trimethoprim, with phenotypic trimethoprim resistance in both isolates and urine. Rapid determination of resistance profiles to first-line antibiotics would avoid unnecessary antibiotic prescription, aid clinical decision making and ultimately improve patient outcomes.

## Materials and methods

### Clinical bacterial isolates

*E. coli* (*n* = 91), *Klebsiella* spp. (*n* = 33), *Proteus mirabilis* (*n* = 61) and *Staphylococcus saprophyticus* (*n* = 17) isolates were from culture-positive urine samples obtained from the Belfast and Northern Health and Social Care Trusts (BHSCT and NHSCT) routine diagnostic microbiology laboratories in October 2014. Isolates were grown on selective agar and identified using the VITEK® 2 system (bioMérieux, Marcy-l’Etoile, France). Following transfer to our laboratory, isolates were re-grown on Tryptone Soy Agar to obtain pure cultures and the identity of the isolates was confirmed by 16S rRNA marker-gene sequencing using primer pairs 27F (5′-AGAGTTTGATCMTGGCTCAG-3′) and 1492R (5′-TACGGYTACCTTGTTACGACTT-3′) [11].

### Antimicrobial susceptibility testing

The antimicrobial susceptibility of *E. coli* and *Klebsiella* spp. isolates (*n* = 124) to amoxicillin, ceftazidime, ciprofloxacin, co-amoxiclav, nitrofurantoin and trimethoprim was determined by ETEST® (bioMérieux, Marcy-l’Etoile, France) according to the manufacturer’s instructions. Susceptibility of *Proteus mirabilis* (*n* = 61) and *S. saprophyticus* (*n* = 17) to trimethoprim was also determined by ETEST®. The isolates were classified as susceptible, intermediate or resistant to each antibiotic according to the European Committee on Antimicrobial Susceptibility Testing (EUCAST) MIC breakpoints [12].

### Detection of trimethoprim resistance (*dfrA*) genes in clinical urinary isolates

The most common trimethoprim-resistance genes in Europe (*dfrA1, dfrA5*, *dfrA7*, *dfrA12* and *dfrA17*) [13, 14], were detected by polymerase chain reaction (PCR) amplification using the Applied Biosystems Veriti™ 96-Well Thermal Cycler (Thermo Fisher Scientific, Paisley, UK), with primers described in Table S1 (see Supplementary material). Genomic DNA was extracted from bacterial isolates using the DNeasy Blood and Tissue Kit (Qiagen®, Hilden, Germany) in accordance with the manufacturer’s instructions and all reactions were performed in uniplex. Based on the high homology between *dfrA7 and dfrA17*, one primer set (*dfrA7/dfrA17)* was designed to detect both genes. The final PCR reaction mixture (50 μL) for *dfrA* genes contained 0.4 μM of each forward and reverse primers (Eurofins MWG Operon, Ebersberg, Germany) and 1 μL of DNA template, with initial denaturation at 95 °C for 2 min; 30 cycles of denaturation at 95 °C for 30 s, annealing at 65 °C (*dfrA1*, *dfrA5*, *dfrA12*) or 55 °C (*dfrA7/17*) for 30 s and extension at 72 °C for 15 s; and a final extension at 72 °C for 5 min. DNA from isolates previously sequenced and confirmed to harbour the *dfrA* genes (*dfrA1* – *E. coli* UM015; *dfrA5* – *E. coli* UM176; *dfrA7/17* – *E. coli* UM107; *dfrA12 - K. pneumoniae* UM282) were used as positive controls while DEPC-treated water (Ambion, Warrington, UK) was used as a negative control. The PCR products were separated by size on a 1.5% (w/v) agarose (Invitrogen, Paisley, UK) gel at 100 V for 30 min.

### Culture-independent detection of trimethoprim resistance in urine

Ninety-four (94) urine samples were collected in May 2019 from the Routine Diagnostic Laboratory, BHSC, with ethical approval (ORECNI Reference: 17/SC/0302). These were samples submitted for routine urine culture and no patient metadata was collected. DNA was immediately extracted from these urine samples on the automated MagNA Pure 96 (Roche, Germany) platform using the DNA and viral NA small volume kit (Roche, Germany), according to the manufacturer’s instructions. The extracted DNA was stored at − 20 °C before use as template in the PCR reaction. Microbiological culture of urine samples was performed on Brilliance UTI Clarity agar (Fannin L.I.P., Galway) and susceptibility testing of the isolates to trimethoprim was determined by ETEST®. The diagnostic performance of *dfrA* for predicting trimethoprim resistance using a culture-independent PCR method was determined and compared with phenotypic trimethoprim resistance.

### Statistical analysis

The Chi-square test was performed to compare the antibiotic susceptibility between *E. coli* and *Klebsiella* spp. Fisher’s exact test was used to determine the association between the presence of *dfrA* genes and trimethoprim resistance. Isolates were grouped into Gram-positive and Gram-negative bacteria for the analysis of the association (Fisher’s exact test) between culture-independent detection of *dfrA* in urine samples and phenotypic trimethoprim resistance. All statistical analyses were carried out using GraphPad Prism 6 for Windows version 6.01 (GraphPad Software Inc., CA, USA). A *P* value *<* 0.05 was considered statistically significant.

## Results

### Antimicrobial susceptibility of *E. coli* and *Klebsiella* spp. isolates

Trimethoprim resistance was phenotypically detected in 37/124 (29.8%) of the *E. coli* and *Klebsiella* spp. isolates. Four of these 37 (10.8%) trimethoprim-resistant isolates were resistant to trimethoprim only, 21/37 (56.8%) were resistant to an additional antibiotic, 10/37 (27%) were resistant to two additional antibiotics, while 2/37 (5.4%) were resistant to three additional antibiotics (Table 1). Data on susceptibility of *E. coli* and *Klebsiella* spp. isolates to all antibiotics tested are summarized in Table S2 (see Supplementary material). Fewer *Klebsiella* spp. isolates (7/33; 21.2%) showed intermediate or resistant phenotypes to trimethoprim than *E. coli* (31/91; 34.1%) isolates, although this difference was not statistically significant (*P* > 0.05; Chi-square test). Eighty-one of the 124 (65.3%) isolates were resistant to amoxicillin while 15/124 (12.1%) isolates were not susceptible to ciprofloxacin (Table 1). Resistance to nitrofurantoin was observed in 18/124 (14.5%) isolates and was more apparent in *Klebsiella* spp. (10/33; 30.3%) than in *E. coli* (8/91; 8.8%) isolates (*P* < 0.01; Chi-square test), with MIC_90_ values of > 512 and 24 mg/L, respectively. Nonetheless, significantly fewer *Klebsiella* spp. isolates were not susceptible to ciprofloxacin (6/33; 18.2%) than nitrofurantoin (10/33; 30.3%) (*P* < 0.05; Chi-square test).

**Table 1:**
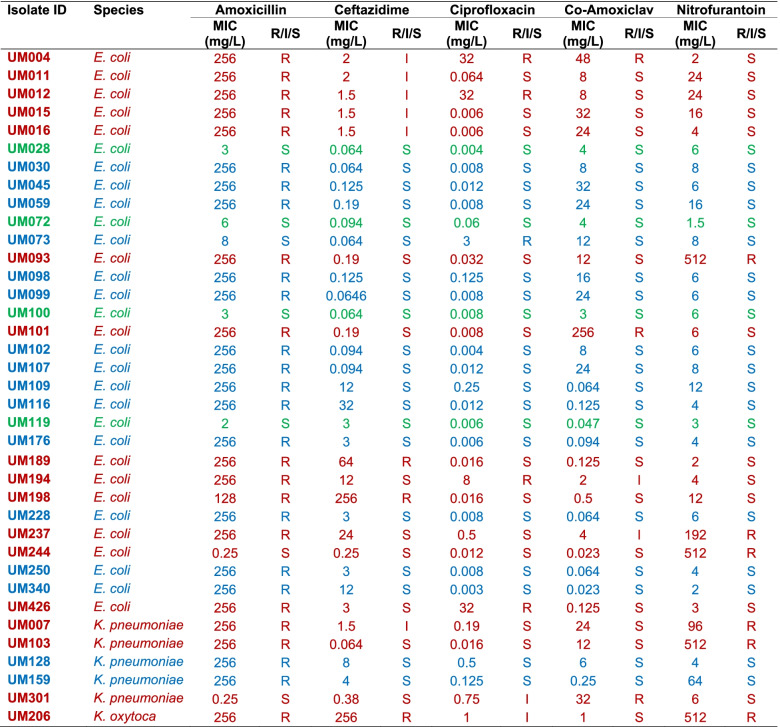
Antimicrobial susceptibility of trimethoprim-resistant *E. coli* and *Klebsiella* spp. isolates

Green: Isolates resistant to trimethoprim only; Blue: Isolates resistant to trimethoprim and an additional antibiotic; Brown: Isolates resistant to trimethoprim and intermediate-resistant to ≥2 antibiotics

*R* Resistant, *I* Intermediate, *S* Susceptible, *MIC* Minimum Inhibitory Concentration

### *dfrA* as a marker for phenotypic trimethoprim resistance in *E. coli* and *Klebsiella* spp.

**Fig. 1:**
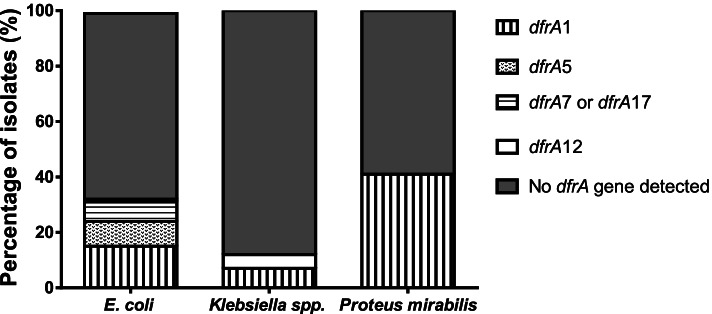
Prevalence of *dfr*A genes in urinary *Escherichia coli* (*n* = 91), *Klebsiella* spp. (*n* = 33) and *Proteus mirabilis* (*n* = 61) isolates

Of the 124 *E. coli* and *Klebsiella* spp. isolates, the *dfrA* gene targets were detected in 31 isolates comprising 28 *E. coli* (*dfrA1*, *n* = 13; *dfrA5, n* = 8; *dfrA7/dfrA17*, *n* = 7) and 3 *Klebsiella* spp. (*dfrA1*, *n* = 2 and *dfrA12,* n = 1) (Fig. 1). Representative gels for the detection of dfrA genes are shown in Fig. S1. Of the 31 trimethoprim-resistant *E. coli* isolates, *dfrA* was present in 27 (87.1%; *dfrA1*, *n* = 12; *dfrA5*, n = 8; *dfrA7&17*, n = 7); in contrast, it was only detected in 2/6 (33.3%; *dfrA1*, n = 2) trimethoprim-resistant *Klebsiella* spp. Furthermore, *dfrA* was detected in only 2/87 (2.3%; *dfrA1*, n = 1 and *dfrA12*, n = 1) trimethoprim-sensitive isolates. There was a significant association between the presence of *dfrA* and trimethoprim resistance among the isolates tested (*P* < 0.0001; Fisher’s exact test). The sensitivity and specificity of *dfrA* detection to determine phenotypic trimethoprim resistance compared to phenotypic susceptibility testing by ETEST® was 93.6 and 91.4% respectively, with a Positive Predictive Value (PPV) of 78.4% and a Negative Predictive Value (NPV) of 97.7%.

### Trimethoprim resistance and *dfrA* detection in other uropathogens

The ability of *dfrA* to predict phenotypic trimethoprim resistance in other common urinary isolates, *P. mirabilis* and *S. saprophyticus,* was also investigated. Thirty-seven of 61 (60.7%) *P. mirabilis* isolates were resistant to trimethoprim (Range: 0.5 - > 32 mg/L; MIC_50_: 2 mg/L; MIC_90_: 6 mg/L) and *dfrA* was detected in 25/37 (67.6%). Only *dfrA1* (25/25) was detected in trimethoprim-resistant *P. mirabilis,* with no *dfrA* genes detected in trimethoprim-sensitive *P. mirabilis* isolates (0/24). There was a significant association between the presence of *dfrA* and trimethoprim resistance among *P. mirabilis* isolates (*P* < 0.0001; Fisher’s exact test) [Sensitivity = 67.6%; Specificity = 100%; PPV = 100%; NPV = 66.7%]. Only 1/17 (5.9%) *S. saprophyticus* isolate was resistant to trimethoprim (MIC: > 32 mg/L) and none of the *dfrA* genes tested in this study were detected in *S. saprophyticus* isolates.

### Culture-independent PCR detection of trimethoprim resistance in urine samples

Of the 94 urine samples tested, 92 (97.9%) were culture-positive and two samples (2.1%) had no detectable bacteria. Of the culture-positive samples, 55 (59.8%) were monomicrobial and 37 (40.2%) were polymicrobial (Table S3). Twenty of the 94 samples (21.3%) were positive by culture for bacteria which were phenotypically resistant to trimethoprim. *DfrA* genes were detected in 15/20 trimethoprim-resistant bacteria. *DfrA* genes were also detected by PCR in 16/94 (17%) urine samples (Table 2). Of these 16 urine samples positive for *dfrA*, *dfrA*-positive bacteria that were phenotypically resistant to trimethoprim were cultured from 15. In one urine sample (CU0000058), *dfrA* was detected by PCR but the isolate cultured from the sample was *dfrA*-negative and susceptible to trimethoprim. *DfrA* genes were not detected by PCR from two urine samples (CU0000042 and CU0000063), which were culture-positive for *dfrA*-positive isolates demonstrating phenotypic resistance to trimethoprim. Three additional urine samples (CU0000025, CU0000027 and CU0000078) with no detectable *dfrA* genes were culture-positive for isolates phenotypically not susceptible to trimethoprim. There was a significant association (*P* < 0.0001; Fisher’s exact test) between the presence of *dfrA* and phenotypic trimethoprim resistance in urine samples containing Gram-negative bacteria (Sensitivity = 75%; Specificity = 96.9%; PPV = 93.8%; NPV = 86.1%).

**Table 2:** Detection of trimethoprim resistance in urine

Sample ID	Culture-independent detection of ***dfrA*** gene(s)	Organisms isolated from urine	***dfrA*** gene(s) detected in isolates	Susceptibility
CU0000025	ND	*Proteus* spp.	ND	R
CU0000027	ND	*Proteus* spp.	ND	I
CU0000031	*dfrA7&17*	*E. coli*	*dfrA7/17*	R
CU0000032	*dfrA5*	*E. coli*	*dfrA5*	R
CU0000036	*dfrA1*	*Proteus* spp.^a^ and *Klebsiella* spp.	*dfrA1*	R
CU0000042	ND	*E. coli*	*dfrA5*	R
CU0000058	*dfrA1*	*E. coli*^a^*; Klebsiella* spp. and *Staphylococcus* spp.	ND	S
CU0000059	*dfrA1; dfrA7&17*	*E. coli*	*dfrA1* and *dfrA7&17*	R
CU0000063	ND	*E. coli*^a^ and *Enterococcus* spp.	*dfrA5*	R
CU0000069	*dfrA1*	*E. coli*^a^ and *Enterococcus* spp.	*dfrA1*	R
CU0000073	*dfrA5*	*E. coli*	*dfrA5*	R
CU0000077	*dfrA7/17*	*E. coli*^a^ and *Enterococcus* spp.	*dfrA7/17*	R
CU0000078	ND	*E. coli*	ND	R
CU0000079	*dfrA5*	*E. coli*^a^ and *Enterococcus* spp.	*dfrA5*	R
CU0000080	*dfrA1*	*E. coli*^a^ and *Enterococcus* spp.	*dfrA1*	R
CU0000081	*dfrA1*	*E. coli*^a^ and *Enterococcus* spp.	*dfrA1*	R
CU0000089	*dfrA1*	*E. coli*^a^ and *Proteus* spp.	*dfrA1*	R
CU0000094	*dfrA1*	*E. coli*^a^ and *Enterococcus* spp.	*dfrA1*	R
CU0000101	*dfrA7&17*	*E. coli*^a^ and *Enterococcus* spp.	*dfrA7/17*	R
CU0000105	*dfrA1*	*Proteus* spp.^a^ and *Enterococcus* spp.	*dfrA1*	R
CU0000111	*dfrA7&17*	*E. coli*^a^ and *Enterococcus* spp.	*dfrA7/17*	R

*Abbreviations*: *R* Resistant, *I* Intermediate, *S* Susceptible

^a^Predominant organism tested for trimethoprim susceptibility

*ND* Not detected

## Discussion

This study demonstrates that phenotypic trimethoprim resistance can be rapidly and reliably predicted by molecular detection of *dfrA* genes in isolates and urine samples. Timely and appropriate initiation of antimicrobial therapy is key to effective antimicrobial stewardship policies. Rapid detection of trimethoprim resistance, prior to prescription of an antibiotic, could help avoid the risk of treatment failure [15], longer hospitalizations and UTI recurrence [16, 17]. Ongoing work by our group is focusing on the development of a point of care assay which will combine molecular detection of both uropathogens and trimethoprim resistance directly from urine. Such an assay could potentially be used in a range of primary and secondary care settings and may help determine potential resistance to antibiotics in a more clinically relevant timeframe which will guide appropriate antibiotic choice, particularly in patients for whom nitrofurantoin is not recommended.

The prevalence of trimethoprim resistance in *E. coli* reported in this study (34.1%) is similar to data reported for England in 2016 (34%) [18]; however, more recent data demonstrates that resistance in *E. coli* has decreased slightly in England (35.1%, 2015 vs. 31.2%, 2018) [19], Scotland (34.3%, 2015 vs. 33.8%, 2018) [20] and Wales (38.2%, 2015 vs. 36.6%, 2018) [21]. This reduction in trimethoprim resistance is associated with a decrease in trimethoprim use and an increase in nitrofurantoin prescribing for treatment of UTIs [19]. Although there is evidence of reduced trimethoprim use in primary care in Northern Ireland [22], there is currently no data with respect to the impact this is having on trimethoprim resistance among urinary isolates.

Despite high trimethoprim resistance rates, the current National Institute for Health and Care Excellence (NICE) guidelines for treatment of UTIs still recommend trimethoprim as first-line treatment in patient cohorts such as adult males ≥16 years old, non-pregnant women ≥16 years old and children aged ≥3 months, where there is low risk of resistance [6]. Results from this study have demonstrated that a PCR-based molecular test can be used to identify the risk of trimethoprim resistance from isolates and from urine without culture. Culture-independent detection of *dfrA* genes in urine results in a more rapid detection of trimethoprim resistance, with a 3–4 hour turnaround time. Furthermore, current phenotypic detection of trimethoprim resistance depends on determining susceptibility of the culture-predominant isolate. However, culture-independent testing of one urine sample in the current study demonstrated the presence of trimethoprim resistance, which was not identified based on phenotypic testing of the culture predominant organism.

In the current study, phenotypic trimethoprim resistance was detected in 37/124 (29.8%) *E. coli* and *Klebsiella* spp. isolates and at least one *dfrA* gene (*dfrA1*, *dfrA5*, *dfrA7*, *dfrA12* or *dfrA17*) was detected in 29/37 (78%) of trimethoprim-resistant isolates tested. This is similar to previous studies that investigated trimethoprim resistance among *E. coli* isolates and detected *dfrA1*, *dfrA5*, *dfrA7*, *dfrA12* and *dfrA17* genes in 75–86% of isolates tested [13, 14]. *DfrA17* was recently identified as a diagnostically relevant AMR biomarker for trimethoprim-sulfamethoxazole resistance through metagenomic screening of over 1000 clinical *E. coli* isolates [23]. The *dfrA* genes investigated in this study were not detected in 20 Gram-negative isolates (4 *E. coli*, 4 *Klebsiella pneumoniae* and 12 *Proteus mirabilis*) which were phenotypically resistant to trimethoprim. It is possible that these isolates harbour one or more of the > 30 different *dfr* genes which have been reported to encode trimethoprim resistance [24] and were not targeted by the PCR assay used in this study.

Nitrofurantoin resistance among *E. coli* isolates in this study (8.8%) is higher than the 3% reported in the first quarter of 2017 in England [18] and 1.8% in Scotland in 2018 [20] but lower than reports from Wales in 2018 (11%) [21]. Lower resistance levels of *E. coli* to nitrofurantoin has also been reported in other European countries [25–27], the United States [28], and Australia [29]. While nitrofurantoin resistance is low in the general population, it has been reported to be higher in specific cohorts such as ≥65-year-old males (22.9%) [19]. The higher resistance rates observed in this study may be because urine samples were submitted to the diagnostic laboratory, based on a clinical suspicion of infection. Similar to previous studies [7, 30, 31], this study observed that nitrofurantoin resistance was higher in *Klebsiella* spp. than in *E. coli* (see Table S2 in Additional file). This highlights the need for increased surveillance of nitrofurantoin resistance among urinary *Klebsiella* spp. isolates, especially as prescribing of nitrofurantoin for UTI treatment is increasing [32].

With the exception of a single isolate with an MIC > 32 mg/L, *S. saprophyticus* isolates in this study were all susceptible to trimethoprim. No *dfrA* gene was detected in any of the *S. saprophyticus* isolates tested, although trimethoprim-resistant isolates have been reported in a previous study [33]. Similarly, no *dfrA* gene was detected by PCR in urine samples containing only Gram-positive organisms, in single and mixed culture. While there is a *dfrA* gene, which confers trimethoprim resistance in *S. saprophyticus* and other *Staphylococcus* spp [33, 34]*,* the gene shares limited homology with the *dfrA* in Gram-negative bacteria, which may explain why *dfrA* genes could not be detected in *S. saprophyticus* isolates and urine in this study.

This study has a number of limitations. Firstly, *dfrA* genes were used to predict trimethoprim resistance and as some isolates showed phenotypic resistance without detectable *dfrA,* it is possible that *dfrA* genes not tested in this study may be encoding trimethoprim resistance in these isolates. Furthermore, this study was limited to *E. coli, Klebsiella* spp., *P. mirabilis* and *S. saprophyticus*. Therefore, further studies would be required to determine whether detection of *dfrA* genes could predict trimethoprim resistance in less frequently isolated Gram-negative urinary pathogens such *Enterobacter* spp.*, Morganella morganii* and *Providencia* spp.

## Conclusion

This study showed that the presence of *dfrA* genes can reliably predict phenotypic trimethoprim resistance in urinary *E. coli, Klebsiella* spp. and *P. mirabilis* isolates and in urine without culture. Culture-independent PCR detection of *dfrA* genes in urine could enable more rapid determination of trimethoprim resistance in urine specimens and guide antibiotic prescribing in patients with a UTI. This could improve antibiotic stewardship and be particularly useful in patients with reduced kidney function where nitrofurantoin use is contra-indicated.

## Supplementary Information

**Additional file 1: Table S1.** Primers used for the detection of *dfrA* genes.

**Additional file 2: Table S2.** Antimicrobial susceptibility of urinary *E. coli* and *Klebsiella* spp. isolates. *Abbreviations: S: Susceptible; I: Intermediate; R: Resistant.* Breakpoints: Amoxicillin (S ≤ 8; R > 8); Ceftazidime (S ≤ 1; I = 1.5 – 4; R > 4); Ciprofloxacin (S ≤ 0.5; I = 0.75 – 1; R > 1); Co-amoxiclav (S ≤ 32; R > 32); Nitrofurantoin (S ≤ 64; R > 64); Trimethoprim (S ≤ 2; I = 3 - 4; R > 4). NA: Not Applicable. Intermediate category not approved for amoxicillin, co-amoxiclav and nitrofurantoin by EUCAST. MIC: minimum inhibitory concentration; MIC_50_ and MIC_90_, MICs that inhibit 50% and 90% of the isolates, respectively.

**Additional file 3: Table S3.** Microorganisms isolated from clinical urine samples.

**Additional file 4: Figure S1.** Gels showing detection of *dfrA1* (a), *dfrA5* (b), *dfrA12* (c) and *dfrA7&17* (d) genes.

## Data Availability

The datasets supporting the conclusions of this article are included within the article and its supplementary materials.

## Abbreviations

UTI: Urinary Tract Infection
HSCT: Health and Social Care Trust
AST: Antimicrobial Susceptibility Testing
EUCAST: European Committee on Antimicrobial Susceptibility Testing
MIC: Minimum Inhibitory Concentration
PCR: Polymerase Chain Reaction
DEPC: Diethyl pyrocarbonate
