# Supplementary material for: Antimicrobial resistance in urinary pathogens and culture-independent detection of trimethoprim resistance in urine from patients with urinary tract infection
Source: BMC Microbiol. 2022 May 24;22:144. doi: 10.1186/s12866-022-02551-9 (PMC9128081; doi:10.1186/s12866-022-02551-9)
Supplement: Supplementary file 1 — Additional file 1: Table S1. Primers used for the detection of dfrA genes. [file 12866_2022_2551_MOESM1_ESM.docx]

**Table S1:** Primers used for the detection of dfrA genes

| Primer name | Primer sequence  (5′ - 3′) | Primer Length (bp) | Tm (°C) | GC content (%) | Amplicon size (bp) |
| --- | --- | --- | --- | --- | --- |
| dfrA1F2 | ACTTTCCTGAAATCCCCAGCAATTTTAG | 28 | 62.2 | 39 | 130 |
| dfrA1R2_RC | GCGTGACAGGTTTGCGAATCCGT | 23 | 64.2 | 57 |  |
| dfrA5F2 | AATGACAACGTAATAGTATTCCCGTCGAT | 29 | 62.4 | 38 | 128 |
| dfrA5R2_RC | AGCGTAGAGGCCATGGGCAATGTT | 24 | 64.4 | 54 |  |
| dfrA7&17F1 | TACTCTTTAAAGCGCTCACATATAATCAGTG | 31 | 62.9 | 36 | 133 |
| dfrA7&17R1_RC | ACATTTTCATTTGAGCTTGAAATTCCTTTCCT | 32 | 61.8 | 31 |  |
| dfrA12F2 | TATCGCTTTGGCATCCGAACTCGG | 24 | 64.4 | 54 | 117 |
| dfrA12R2_RC | AAGGTTTGATGTACCTCAGATAGAAACAC | 29 | 62.4 | 38 |  |
