# Supplementary material for: Antimicrobial resistance in urinary pathogens and culture-independent detection of trimethoprim resistance in urine from patients with urinary tract infection
Source: BMC Microbiol. 2022 May 24;22:144. doi: 10.1186/s12866-022-02551-9 (PMC9128081; doi:10.1186/s12866-022-02551-9)
Supplement: Supplementary file 2 — Additional file 2: Table S2. Antimicrobial susceptibility of urinary E. coli and Klebsiella spp. isolates. Abbreviations: S: Susceptible; I: Intermediate; R: Resistant. Breakpoints: Amoxicillin (S ≤ 8; R > 8); Ceftazidime (S ≤ 1; I = 1.5 – 4; R > 4); Ciprofloxacin (S ≤ 0.5; I = 0.75 – 1; R > 1); Co-amoxiclav (S ≤ 32; R > 32); Nitrofurantoin (S ≤ 64; R > 64); Trimethoprim (S ≤ 2; I = 3 - 4; R > 4). NA: Not Applicable. Intermediate category not approved for amoxicillin, co-amoxiclav and nitrofurantoin by EUCAST. MIC: minimum inhibitory concentration; MIC50 and MIC90, MICs that inhibit 50% and 90% of the isolates, respectively. [file 12866_2022_2551_MOESM2_ESM.docx]

**Table S2**: Antimicrobial susceptibility of urinary E. coli and Klebsiella spp. isolates

| Antibiotic | MIC (mg/L) for all isolates | | |  | | Number (Percentage) of isolates with indicated susceptibilities | | |
| --- | --- | --- | --- | --- | --- | --- | --- | --- |
|  | Range | MIC_50_ | MIC_90_ |  | S | | I | R |
| *E. coli* (n=91) |  |  |  |  |  | |  |  |
| Trimethoprim | 0.094 - >32 | 0.5 | >32 |  | 60 (65.9) | | 0 | 31 (34.1) |
| Amoxicillin | 0.25 - >256 | >256 | >256 |  | 40 (44) | | NA | 51 (56) |
| Ceftazidime | 0.0064 - 6 | 0.094 | 2 |  | 76 (83.5) | | 14 (15.4) | 1 (1.1) |
| Ciprofloxacin | 0.002 – >32 | 0.008 | 0.25 |  | 84 (92.3) | | 0 | 7 (7.7) |
| Co-amoxiclav | 0.25 - >256 | 8 | 128 |  | 75 (82.4) | | NA | 16 (17.6) |
| Nitrofurantoin | 0.19 - >512 | 8 | 24 |  | 83 (91.2) | | NA | 8 (8.8) |
|  |  |  |  |  |  | |  |  |
| *Klebsiella* spp. (n=33) |  |  |  |  |  | |  |  |
| Trimethoprim | 0.094 - >32 | 0.5 | >32 |  | 26 (78.8) | | 1 (3.0) | 6 (18.2) |
| Amoxicillin | 0.25 - >256 | 128 | >256 |  | 3 (9.1) | | NA | 30 (90.9) |
| Ceftazidime | 0.032 - 32 | 0.094 | 2.7 |  | 26 (78.8) | | 4 (12.1) | 3 (9.1) |
| Ciprofloxacin | 0.006 – 1 | 0.023 | 0.5 |  | 30 (81.8) | | 3 (9.1) | 0 |
| Co-amoxiclav | 0.38 - >256 | 3 | 15.2 |  | 31 (93.9) | | NA | 2 (6.1) |
| Nitrofurantoin | 2 - >512 | 16 | >512 |  | 23 (69.7) | | NA | 10 (30.3) |
|  |  |  |  |  |  | |  |  |
| Overall (n=124) |  |  |  |  |  | |  |  |
| Trimethoprim | 0.094 - >32 | 0.5 | >32 |  | 86 (69.4) | | 1 (0.8) | 37 (29.8) |
| Amoxicillin | 0.25 - >256 | >256 | >256 |  | 43 (34.7) | | NA | 81 (65.3) |
| Ceftazidime | 0.0064 - 32 | 0.094 | 2.7 |  | 102 (82.3) | | 18 (14.5) | 4 (3.2) |
| Ciprofloxacin | 0.002 – >32 | 0.012 | 0.5 |  | 114 (91.9) | | 3 (2.4) | 7 (5.7) |
| Co-amoxiclav | 0.25 - >256 | 4 | 96 |  | 106 (85.5) | | NA | 18 (14.5) |
| Nitrofurantoin | 0.19 - >512 | 8 | 128 |  | 106 (85.5) | | NA | 18 (14.5) |

Abbreviations: S: Susceptible; I: Intermediate; R: Resistant.

Breakpoints: Amoxicillin (S ≤ 8; R > 8); Ceftazidime (S ≤ 1; I = 1.5 – 4; R > 4); Ciprofloxacin (S ≤ 0.5; I = 0.75 – 1; R > 1); Co-amoxiclav (S ≤ 32; R > 32); Nitrofurantoin (S ≤ 64; R > 64); Trimethoprim (S ≤ 2; I = 3 - 4; R > 4).

NA: Not Applicable. Intermediate category not approved for amoxicillin, co-amoxiclav and nitrofurantoin by EUCAST.

MIC: minimum inhibitory concentration; MIC_50_ and MIC_90_, MICs that inhibit 50% and 90% of the isolates, respectively
