# Supplementary material for: Antimicrobial resistance in urinary pathogens and culture-independent detection of trimethoprim resistance in urine from patients with urinary tract infection
Source: BMC Microbiol. 2022 May 24;22:144. doi: 10.1186/s12866-022-02551-9 (PMC9128081; doi:10.1186/s12866-022-02551-9)
Supplement: Supplementary file 3 — Additional file 3: Table S3. Microorganisms isolated from clinical urine samples. [file 12866_2022_2551_MOESM3_ESM.docx]

**Table S3: Microorganisms isolated from clinical urine**

| **Sample ID** | **Organisms** | | |
| --- | --- | --- | --- |
| CU0000021 | *Enterococcus faecalis* | | |
| CU0000022 | *Proteus mirabilis* | | |
| CU0000023 | *Staphylococcus* spp. | | |
| CU0000024 | *Escherichia coli* | | |
| CU0000025 | *Proteus* spp. | | |
| CU0000026 | *Enterococcus faecalis* | | |
| CU0000027 | *Proteus mirabilis* | | |
| CU0000028 | *Escherichia coli* | | |
| CU0000029 | *Escherichia coli* | | |
| CU0000030 | *Enterococcus faecalis* | | |
| CU0000031 | *Escherichia coli* | | |
| CU0000032 | *Escherichia coli* | | |
| CU0000033 | *Proteus mirabilis* | | |
| CU0000034 | *Pseudomonas aeruginosa* | |  |
| CU0000035 | *Enterococcus faecium, Staphylococcus* spp. | | |
| CU0000036 | *Proteus* spp., *Enterococcus* spp. *Klebsiella* spp. | | |
| CU0000037 | *Escherichia coli* | | |
| CU0000038 | *Enterococcus faecalis, Acinetobacter baumannii* | | |
| CU0000039 | *Streptococcus agalactiae* | | |
| CU0000040 | *Enterococcus faecalis* | | |
| CU0000041 | *Citrobacter koseri, Enterococcus faecalis, Morganella morganii* | | |
| CU0000042 | *Escherichia coli* | | |
| CU0000043 | No growth | | |
| CU0000044 | *Klebsiella pneumoniae* | | |
| CU0000045 | *Pseudomonas aeruginosa* | | |
| CU0000046 | *Pseudomonas aeruginosa, Enterococcus* spp*., Escherichia coli* | | |
| CU0000047 | *Enterococcus faecalis* | | |
| CU0000048 | *Streptococcus* spp. | | |
| CU0000049 | *Enterococcus faecalis* | | |
| CU0000050 | *Proteus* spp., *Staphylococcus* spp. | | |
| CU0000051 | *Staphylococcus epidermidis* | | |
| CU0000052 | *Enterococcus* spp. | | |
| CU0000053 | *Staphylococcus aureus* |  |  |
| CU0000054 | *Enterococcus faecalis* | | |
| CU0000055 | No growth | | |
| CU0000056 | *Enterococcus* spp., *Staphylococcus* spp. | | |
| CU0000057 | *Pseudomonas* spp. | | |
| CU0000058 | *Escherichia coli, Klebsiella* spp., *Staphylococcus* spp., *Morganella morganii* | | |
| CU0000059 | *Escherichia coli* | | |
| CU0000060 | *Pseudomonas aeruginosa, Staphylococcus* spp. | | |
| CU0000061 | *Enterococcus* spp., *Escherichia coli* | | |
| CU0000062 | *Escherichia coli* | | |
| CU0000063 | *Escherichia coli, Enterococcus* spp. | | |
| CU0000064 | *Escherichia coli, Enterococcus* spp. | | |
| CU0000065 | *Pseudomonas aeruginosa* | | |
| CU0000066 | *Enterococcus faecalis* | | |

**Table S3 (continued): Microorganisms isolated from clinical urine**

| **Sample ID** | **Organisms** |
| --- | --- |
| CU0000067 | *Escherichia coli* |
| CU0000068 | *Enterococcus faecalis* |
| CU0000069 | *Escherichia coli, Enterococcus* spp. |
| CU0000070 | *Escherichia coli* |
| CU0000071 | *Escherichia coli, Enterococcus* spp. |
| CU0000072 | *Escherichia coli* |
| CU0000073 | *Escherichia coli* |
| CU0000074 | *Escherichia coli* |
| CU0000075 | *Escherichia coli, Enterococcus* spp. |
| CU0000076 | *Enterococcus faecalis* |
| CU0000077 | *Escherichia coli, Enterococcus* spp. |
| CU0000078 | *Escherichia coli* |
| CU0000079 | *Escherichia coli, Enterococcus faecalis* |
| CU0000080 | *Escherichia coli, Enterococcus* spp. |
| CU0000081 | *Escherichia coli, Enterococcus* spp. |
| CU0000082 | *Klebsiella* spp. |
| CU0000083 | *Escherichia coli* |
| CU0000084 | *Proteus mirabilis* |
| CU0000085 | *Enterococcus faecalis* |
| CU0000086 | *Proteus mirabilis* |
| CU0000087 | *Escherichia coli, Enterococcus faecalis* |
| CU0000088 | *K. pneumoniae* |
| CU0000089 | *Escherichia coli, Proteus* spp. |
| CU0000090 | *Enterococcus faecalis* |
| CU0000091 | *Escherichia coli, Enterococcus faecium* |
| CU0000092 | *Enterococcus faecalis* |
| CU0000093 | *Enterococcus faecalis* |
| CU0000094 | *Escherichia coli, Enterococcus* spp. |
| CU0000095 | *Klebsiella pneumoniae* |
| CU0000096 | *Escherichia coli* |
| CU0000097 | *Escherichia coli* |
| CU0000098 | *Escherichia coli* |
| CU0000099 | *Escherichia coli, Enterococcus* spp., *Proteus* spp. |
| CU0000100 | *Enterococcus* spp., *Streptococcus agalactiae* |
| CU0000101 | *Escherichia coli, Enterococcus spp.* |
| CU0000102 | *Escherichia coli, Enterobacter cloacae* |
| CU0000103 | *Enterococcus* spp., *Proteus* spp. |
| CU0000104 | *Escherichia coli, Enterococcus spp.* |
| CU0000105 | *Klebsiella oxytoca, Proteus mirabilis* |
| CU0000106 | *Enterococcus spp., Staphylococcus saprophyticus* |
| CU0000107 | *Enterococcus spp., Streptococcus* spp. |
| CU0000108 | *Klebsiella* spp., *Enterococcus* spp. |
| CU0000109 | *Citrobacter koseri* |
| CU0000110 | *Klebsiella* spp. |
| CU0000111 | *Escherichia coli, Enterococcus* spp*.* |
| CU0000112 | *Klebsiella* spp., *Enterococcus* spp. |
| CU0000113 | *Escherichia coli, Citrobacter* spp. |
| CU0000114 | *Escherichia coli, Enterococcus* spp*.* |
