# Supplementary figures and images for: Antimicrobial resistance in urinary pathogens and culture-independent detection of trimethoprim resistance in urine from patients with urinary tract infection
Source: BMC Microbiol. 2022 May 24;22:144. doi: 10.1186/s12866-022-02551-9 (PMC9128081; doi:10.1186/s12866-022-02551-9)

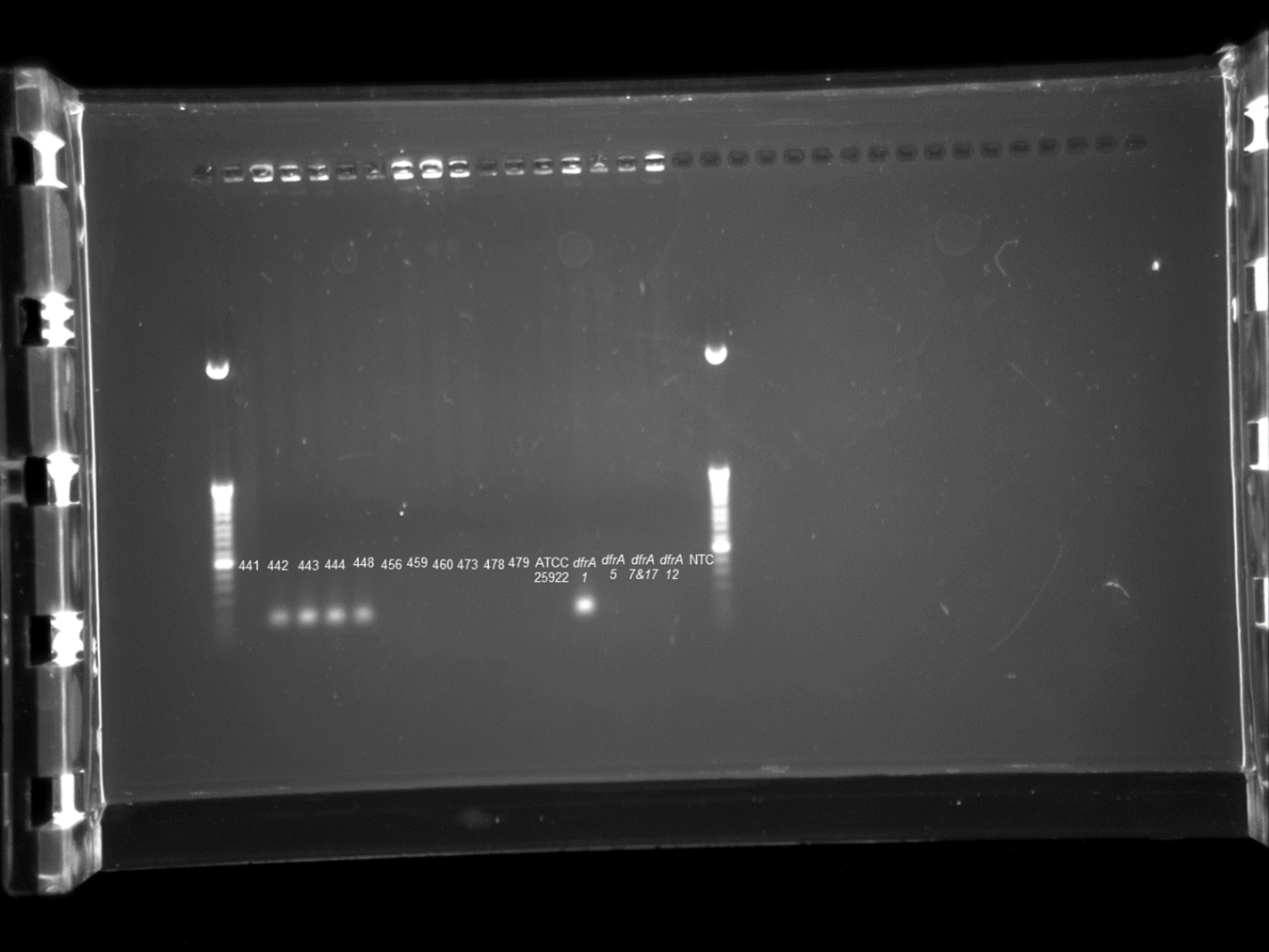


**a**

**b**


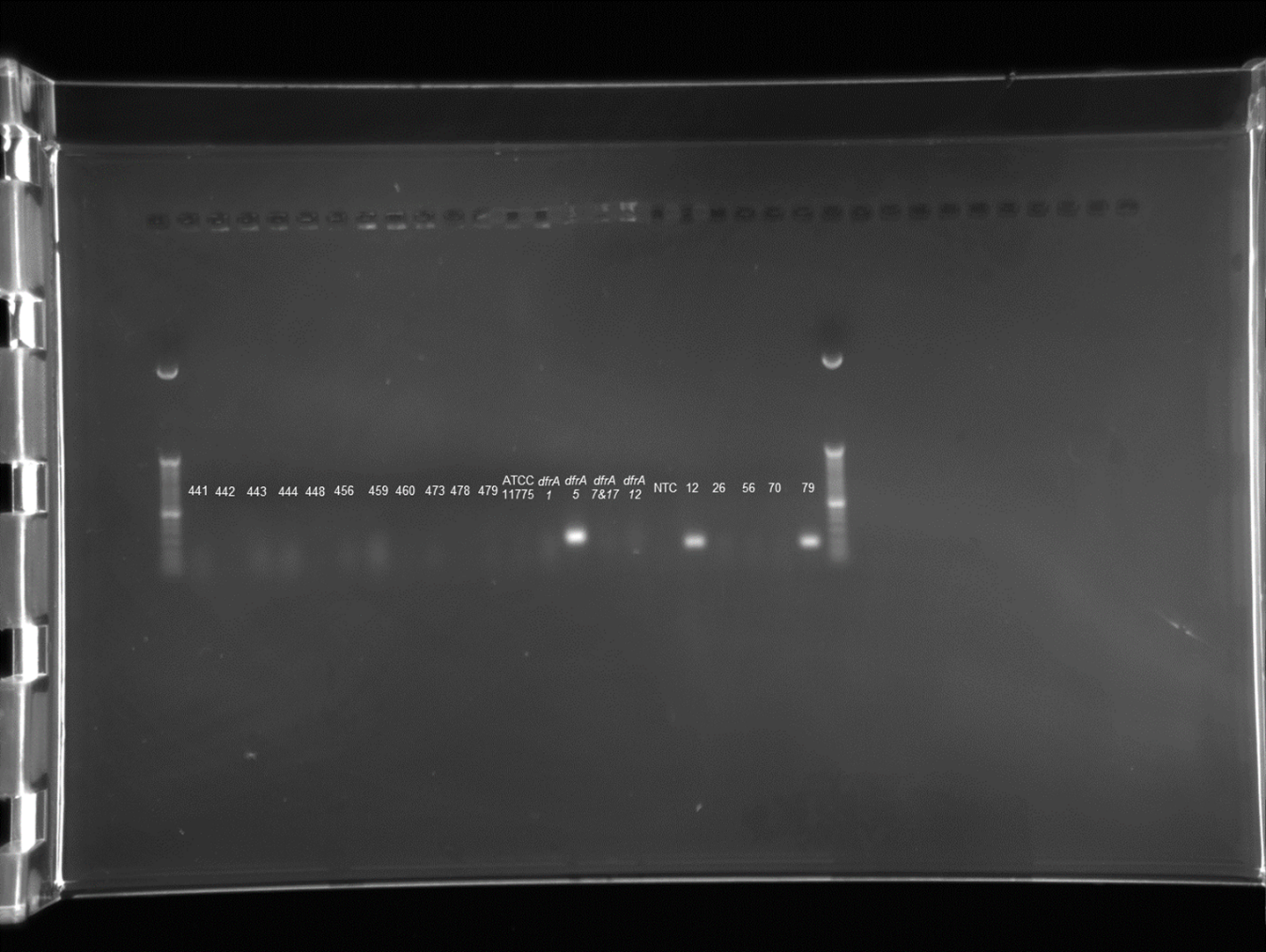


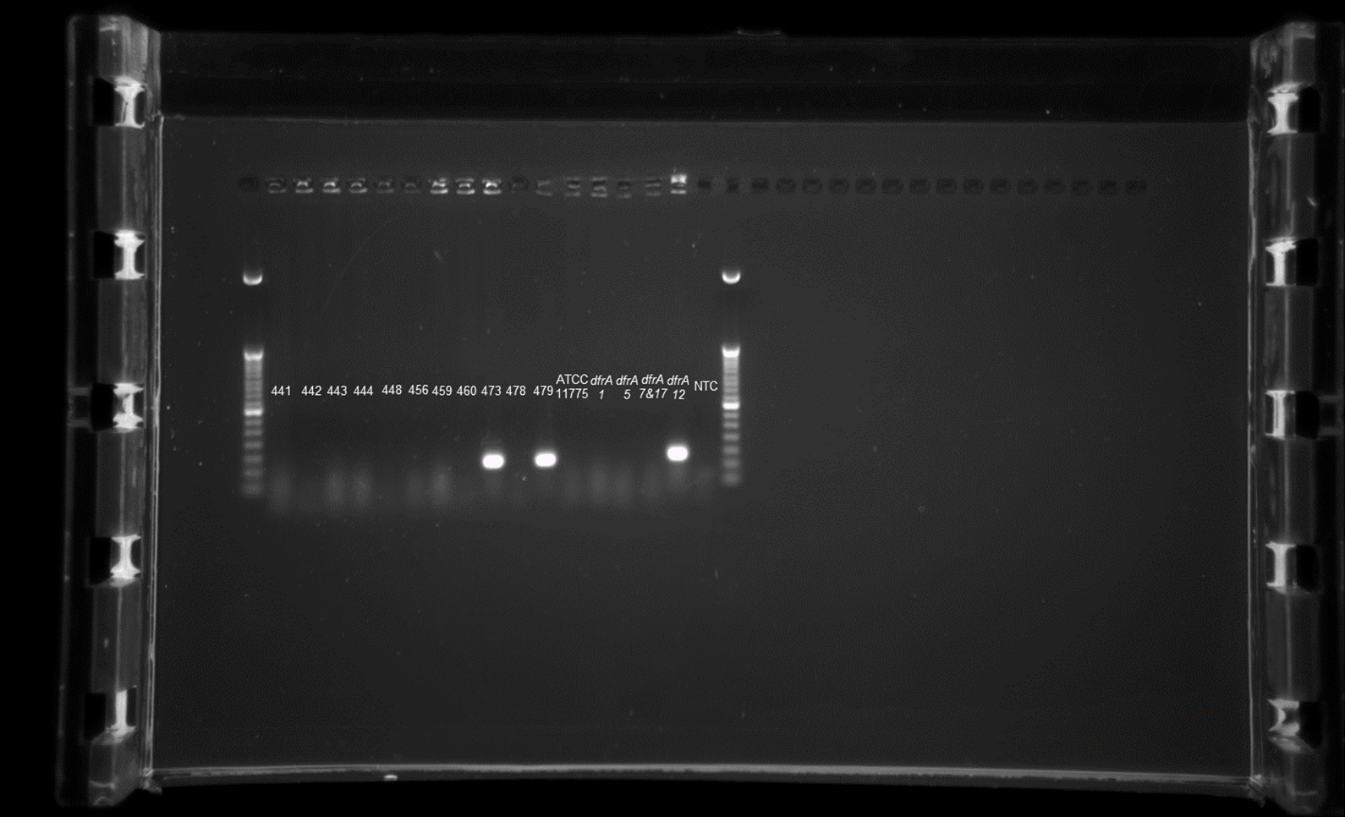


**c**

**d**

**
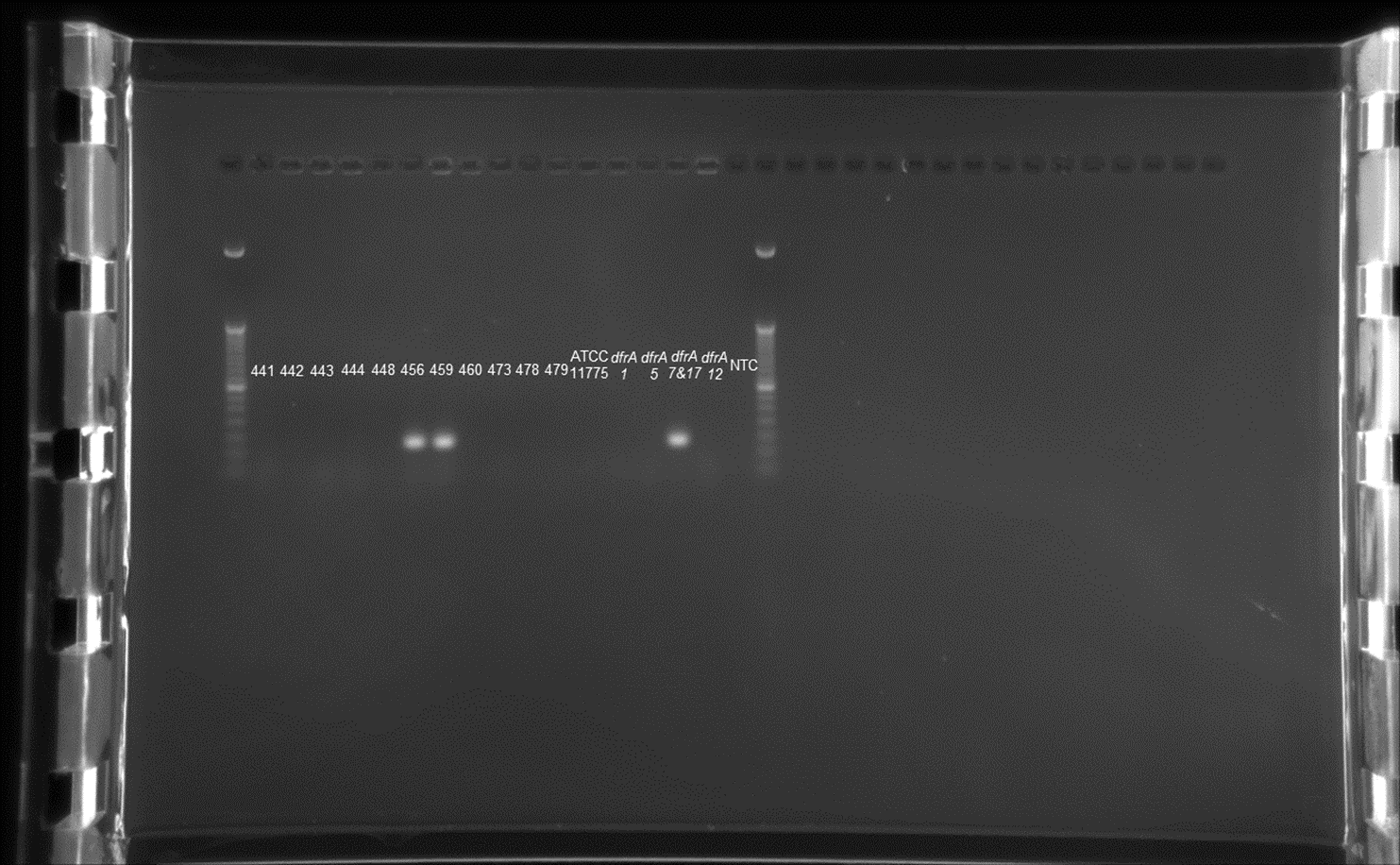
**

**Fig S1**: Gels showing detection of *dfrA1* (a), *dfrA5* (b), *dfrA12* (c) and *dfrA7&17* (d) genes.

Supplement: Supplementary file 4 — Additional file 4: Figure S1. Gels showing detection of dfrA1 (a), dfrA5 (b), dfrA12 (c) and dfrA7&17 (d) genes. [file 12866_2022_2551_MOESM4_ESM.docx]
